# A new handover protocol between old age admission and rehab wards

**DOI:** 10.1192/bjo.2021.92

**Published:** 2021-06-18

**Authors:** Aileen Carmen, Thanurshan Mahenthran, Jemma Hazan, Ioana Popescu

**Affiliations:** Barnet Enfield and Haringey Mental Health Trust

## Abstract

**Aims:**

Efficient handovers are integral to patient care. Challenges to handover for wards include high patient turnover and varied handover approaches between wards, as well as admissions out of hours. Patients on Old Age Wards often have multiple comorbidities and can deteriorate rapidly without coordinated care. Our focus was on improving handover of patients transferred between the Old Age Admissions Ward and Rehabilitation Ward. We aimed to create a ward handover protocol to improve compliance with documenting a pretransfer plan and ensure there was an 80% compliance with completing this plan within 3 months.

**Method:**

An MDT discussion took place in order to explore change ideas. Questionnaires were filled out post implementation of protocol. A handover proforma was designed to capture important patient data and continuing plans. A PDSA cycle was designed to deliver a structured handover.

Per patient measures were collected including: whether a handover took place, recording of current medical and psychiatric issues, documentation of plan and was the plan put into action or reviewed.

MDT feedback was collected on satisfaction with the protocol and handover process using open questions and Likert scale.

**Result:**

Prior to the establishment of the proforma there was no verbal or written handover between wards. In 28% of cases prior to the intervention, blood results were checked and medication reviews took place within the timeframe written in the patient's notes. A proforma was initiated and used for 93% of patient handovers between wards. Blood results were checked according to the planned timeframe in 86% of cases. Where the handover proforma took place, 100% of patients had a medications review. Qualitative detail revealed that key patient appointments such as MRI Brain scans and important plans such as fluid restriction limits were missed before implementing the protocol. Aftewards complex patient plans were recorded and implemented accordingly.

Questionnaire feedback was positive and MDT found the proforma to be helpful and to improve patient safety.

**Conclusion:**

The team viewed the new handover pathway as a positive patient safety tool. Compliance with completing the protocol in the longer term and maintaining change is an area for ongoing improvement.

